# Characterization of bird formations using fuzzy modelling

**DOI:** 10.1098/rsif.2022.0798

**Published:** 2023-02-15

**Authors:** Elisa Perinot, Johannes Fritz, Leonida Fusani, Bernhard Voelkl, Marco S. Nobile

**Affiliations:** ^1^ Konrad Lorenz Institute of Ethology, Department of Interdisciplinary Life Sciences, University of Veterinary Medicine, Vienna 1160, Austria; ^2^ Waldrappteam Conservation and Research, 6162 Mutters, Austria; ^3^ Department of Behavioural and Cognitive Biology, University of Vienna, Vienna 1010, Austria; ^4^ Animal Welfare Division, University of Bern, 3012 Bern, Switzerland; ^5^ Department of Environmental Sciences, Informatics and Statistics, Ca’ Foscari University of Venice, 30123 Venezia, Italy; ^6^ Bicocca Bioinformatics, Biostatistics and Bioimaging research center (B4), 20900 Monza, Italy

**Keywords:** fuzzy modelling, collective motion, *Geronticus eremita*, flock behaviour, line formation

## Abstract

The investigation of the emergent collective behaviour in flying birds is a challenging task, yet it has always fascinated scientists from different disciplines. In the attempt of studying and modelling line formation, we collected high-precision position data of 29 free-flying northern bald ibises (*Geronticus eremita*) using Global Navigation Satellite System loggers, to investigate whether the spatial relationships within a flock can be explained by birds maintaining energetically advantageous positions. Specifically, we exploited domain knowledge and available literature information to model by means of fuzzy logic where the air vortices lie behind a flying bird. This allowed us to determine when a leading bird provides the upwash to a following bird, reducing its overall effort. Our results show that the fuzzy model allows to easily distinguish which bird is flying in the wake of another individual, provides a clear indication about flying flock dynamics and also gives a hint about birds' social relationships.

## 1. Background

Some animal species form and move in groups and can achieve astonishing complexity and coordination in space and time, a phenomenon that is defined as collective motion. Well-known examples of collective motion in the animal kingdom are fish schools, herds of herbivores, bird flocks and insect swarms [1]. In the last decades, these behaviours have been studied and modelled extensively not only by biologists but also by computer scientists, physicists, and mathematicians, inspiring the development of a variety of computational intelligence meta-heuristic algorithms such as particle swarm optimization [2], artificial bee colony [3], ant colony optimization [4] or salp swarm optimization [5].

When focusing on flocking behaviour, in the literature we can find a distinction between ‘*cluster formations*’ and ‘*line formations*’ [6,7]. *Cluster formations* refer to three-dimensional groups of birds—generally of small size—flying closely to each other. This is the case of starlings [8], pigeons [9] or even mixed-species flocks [10]. For a long time, scientists have been studying this type of flocking behaviour, trying to figure out *how* birds manage this high level of coordination and cohesion without colliding [11,12], especially during turning, landing, taking off or under predation [13,14].

Instead, *line formations* refer to birds flying in an organized manner that resembles a line or a V and are roughly two-dimensional. Also in this case, scientists' interest fell on how birds achieved this type of synchronization [15,16], although they mostly focused on *why* they behave in this way. They hypothesized that it might be due to the aerodynamic advantage and the energy saving that results from this type of flocking behaviour [6,16–18]. In fact, when a bird is flying and flapping the wings, it generates two regions of upwash in correspondence of the wingtips, that is, the terminal part of the wings. Following individuals can exploit these air vortices to reduce the amount of energy needed when flying. However, in order to do so, a following bird must be displaced laterally and posteriorly with respect to the leading individual with one of its wingtips partly overlapping with the leader's wingtip [19]. Whenever a bird flies in this ‘right position’, where it gains an aerodynamic advantage, it is said to be flying *in-wake*. In addition to the upwash, the frontal bird also produces a downwash, a downward movement of air, which extends directly behind the birds' body and the follower should avoid as it would hinder its flying (see [6] for additional information).

Former studies on cluster formations suggested that birds inside a flock behave similarly to a system of self-propelled particles. In this artificial system, the single units tend to the same average direction of movement of the other neighbouring particles within a certain radius. In other words, particles that are near each other move together, ultimately leading to the emergence of collective behaviour [20]. Similarly, flocking behaviour emerges from the ability of every single bird to self-organize with the closest individuals around it, with no centralized control [11,12,21,22].

To better study flocking behaviour, scientists consequently focused on local or pairwise interactions among the birds, an approach referred to as ‘nearest neighbour approach’ [8,11,20,21,23,24]. Specifically, previous evidence proposed that individuals in a flock interact with a fixed number of neighbours (topological distance) rather than with all the individuals present in a determined radius (metric distance) [8,11,12].

A very similar approach has been used when inferring about line formation and energy savings. In this case, the front-facing closest individual—with respect to Euclidean distance—is assumed to be the one that provides the upwash (as for example in [25,26]). However, we argue that this assumption might be unrealistic and misleading. For a bird to exploit the upwash, the spatial relationship with regard to the bird in the front is more important than simple proximity. For example, a bird might fly behind another individual, so that their bodies are aligned. The two birds are near, but the follower is placed in the downwash, which should instead be avoided for aerodynamic purposes.

An alternative procedure consists in modelling in-wake regions, with fixed size and ‘crisp’ borders, which extend behind the leading bird at the level of the wingtips. In this case, the exact extension and placement of the in-wake areas is required [17,27].

However, the upwash and downwash do not have sharp boundaries, but these air vortices decrease in strength gradually, according to fluid dynamics, i.e. they are intrinsically fuzzy. Thus, we are dealing with imprecise properties and uncertain knowledge about the air vortices and in-wake flying that can be effectively described by means of fuzzy logic. Specifically, we propose a novel approach to develop a knowledge-based model of in-wake flying using fuzzy logic. Fuzzy logic is a special form of multi-valued logic, in which elements can belong to multiple sets with varying degrees of membership (for more information on fuzzy logic, please refer to §2.3 in the methods). We exploited this possibility to build a fuzzy inference system (FIS)—created according to literature data—that is used to determine which positions provide an upwash, with a smooth transition to the regions that do not correspond to in-wake flying.

Fuzzy logic has been employed previously to study flocking behaviour and self-organization in cluster flocks [7,28], but was never applied to the investigation of *line formations*. Our model, introduced in [29], differs from previous attempts as it does not focus on explicitly modelling the birds' behaviour, but it models the upwash (i.e. in-wake flying) and downwash according to a bird's position using fuzzy logic. It comprises information from past literature on flocking behaviour, and it is then applied to empirical data collected from a free-flying flock of northern bald ibises (*Geronticus eremita*).

We hypothesize that a FIS based on three linguistic variables, one for each spatial dimension, could help to model the spatial distribution of the wingtip vortices produced by the frontal bird and therefore allow to identify in-wake flying. We compare our model with the conventional nearest neighbour method, highlighting the differences between the two approaches. We predict that the two models' solutions will differ, as the nearest neighbour approach is based only on proximity and does not take into consideration vortex placement. Besides identifying times when birds are flying in the wake of another bird, the model will allow us to describe flock structure or joint formations (i.e. any subgroup of birds, which are connected by either flying in the wake area of another group member or providing such a wake for another group member). In addition, it will allow us to determine how many of those subgroups exist at any time as well as their size and composition. Finally, it will give the possibility to look into flock spatial dynamics, i.e. how individuals move inside the formation, and social dynamics, i.e. how individuals interact in the formation. We apply the model to an empirical dataset to show how the model works and can be used to draw conclusions on the dynamics of line formation.

## 2. Methods

### 2.1. Data collection

We collected the data in August 2019 during the human-led migration of northern Bald Ibis (*Geronticus eremita*) led by Waldrappteam Conservation and Research (LIFE biodiversity project - LIFE + 12-BIO_AT_000143).^1^ This project aims to reintroduce this species in the wild by imprinting a group of young birds on human foster parents and teaching them to follow a motorized microlight plane with the aim of guiding them through the migration route (see [30] for more details).

Across several flights, each bird was equipped with high-precision custom-made Global Navigation Satellite System (GNSS) loggers (main module NEO-M8T, U-blox AG, Switzerland, *ca* 6L × 2.5W × 4H cm), which fetched the signal from GPS, GLONASS and Galileo satellites, and store all data in a raw format in a SD memory card. The sampling frequency for positioning data was set to 5 Hz. The loggers were secured to the birds using a leg-loop harness with a plate and a custom three-dimensional-printed case, which was designed with an aerodynamic shape and profile to reduce air drag [31,32]. Loggers were mounted on the birds at the beginning of the flight and retrieved at the end, to download the data. During data collection, birds weighed on average 1291 g (range 1138–1456 g), loggers weighed 19.16 ± 0.16 g, and the housing 7–8 g. Therefore, the heaviest logger with the housing weighed 27.32 g, which represents less than 3% of the smallest bird body weight [33]. In addition to the birds, also the microlight was equipped with a logger for reference. An important aspect to highlight is that, although the birds and the microlight flew together, the birds were seldom close to the aircraft and therefore they were not subjected to its aerodynamic influence (mean distance bird–microlight: 30 ± 3.6 m over the 10 min flight) [16,17].

To assess the performance of the modelling approach, we will present a proof-of-principle of our model on a 10 min flight of a flock with 29 birds, collected on 9 August 2019. We selected the central part of the flight because it is more likely that birds fly cohesively as a flock and show flight formation without any interruptions nor landings (please refer to electronic supplementary material for further information).

### 2.2. Data processing

After data collection, we post-processed the data using RTKlib (version demo5 b33b) [34,35] and Python (version 3.7.9). The software RTKlib supports post-processing kinematics (PPK) that calculates the position in the space with very high accuracy. Specifically, normal GNSS (mostly GPS) receivers calculate the position using instantaneous trilateration and can achieve metre-level accuracy (approx. 1–5 m), while PPK achieves cm-level accuracy (i.e. 0.1–10 cm) [36] (please refer to electronic supplementary material for more information). In order to do so, the support of a stationary base station is necessary, which should be as close as possible to the place of data collection. On the day of the data collection, the birds flew in the region around the city of Heiligenberg (Germany). Because of that, we selected the base station PFA300AUT in Bregenz (Austria). This station is part of the EUREF Permanent Network [37] and the high-quality GNSS data continuously collected by those stations is openly accessible.^2^

Using RTKpost (part of RTKlib), we exploited the base station to calculate first the position of the microlight, which then was used as a reference to extract the position of the birds in the flock. We chose this approach to reduce the error of the positioning calculation for the birds, given the medium-distance baseline present between the data collection place and the base location. The output of this procedure was a file containing a series of timestamps and the associated three-dimensional positions of all birds in the flock. After that, we calculated relative positions of the birds with respect to their flight direction as follows.

**Figure 1.**
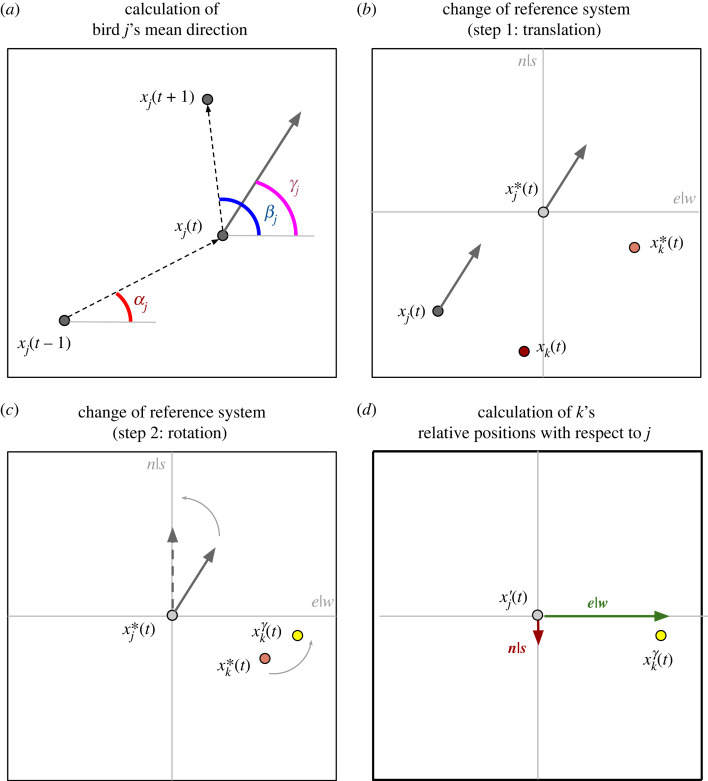
Schematization of the calculation of flying directions. (*a*) How the flight direction of bird *j* is calculated using the positions at timestamps *t* − 1, *t* and *t* + 1. The *α_j_* (in red) and *β_j_* (in blue) angles are calculated and averaged to obtain the angle *γ_j_* (in purple) and the new direction, depicted by the grey arrow. In (*b*), bird *j* is put at the centre of the reference system (translation—from the dark grey to the light grey dot). Also the position of bird *k* is translated relative to bird *j* (from the red to orange dot). The rotation takes place in (*c*), where the flight direction of bird *j* is set to be always towards north, shown with the dashed grey arrow. Together, also rotates bird *k*, as shown with the orange to yellow dot. Finally, in (*d*), we can calculate the relative position of *k* with respect to *j* on the *e*|*w* and *n*|*s* baselines.

We define as $x_{i}(t)\in R^{3}$ the absolute coordinates of *i*-th bird at timestamp *t*, with *i* = 1, …, *N* where *N* denotes the number of birds in the flock. Given a pair of birds (*j*, *k*) (with *j* ≠ *k*), we can calculate the relative flying direction of *k* with respect to *j* during every snapshot *t* > 2 using the procedure schematized in figure 1:

— we use *j*'s positions at time *t* − 1, *t* and *t* + 1 to calculate a mean flight direction. Specifically, we calculated the arctangent of the vectors defined by x*_j_*(*t*) − x*_j_*(*t* − 1) and x*_j_*(*t* + 1) − x*_j_*(*t*). The result is two angles, *α* and *β*, which are then averaged to obtain *γ* (figure 1*a*). We exploited three consecutive positions to filter noise in the data and obtain a smoothed flying path;
— we translated the coordinates of bird *k*, so that bird *j* is centred in the origin of axes (figure 1*b*). We denote by x′ the new position of a bird after such translation;
— we applied a rotation to the coordinates of bird *k*, so that bird *j*'s flying direction becomes aligned to the *y*-axis (figure 1*c*). We denoted by x″ the new position of a bird after such rotation;
— we used the new system of coordinates to compute the relative positioning of bird *k* with respect to *j* (as shown in figure 1*d*). We denoted the relative positioning using a set of two values indicating the east|west and north|south (hereafter shortened as *e*|*w*, *n*|*s*, respectively);
— finally, we included the third dimension, defined up|down (*u*|*d* hereafter), and calculated the relative distance of bird *k* with respect to *j* by subtracting the *u*|*d* position of bird *k* from the *u*|*d* position of bird *j*.

Using this relative spatial information, it is possible to calculate what we define as the frontal nearest neighbour (FNN) as follows. Given a bird *k* in the flock, in each timestamp, the FNN of *k* is defined as the nearest individual *j* (with respect to the Euclidean distance) satisfying the following conditions:

— (i) *j* is leading (i.e. *n*|*s* > 0),
— (ii) it is co-planar (i.e. − 0.75〈*u*|*d* < 0.75, where 0.75 m corresponds to half a wingspan), and
— (iii) not further away than 6 m from the bird of interest (i.e. $\sqrt{{\left(n|s\right)}^{2}+{\left(e|w\right)}^{2}}<=6.0$).

### 2.3. Fuzzy modelling and inference

Fuzzy set theory was introduced by Zadeh [38] as an extension of classic set theory, where an element can belong to one or more disjoint sets with gradual levels of membership. This notion is radically different with respect to classical set theory, in which an element can either belong or not belong to a given set. By means of fuzzy sets, it is possible to formally model vague and uncertain concepts.

Formally, a fuzzy set is defined by a pair (*U*, *m*) where *U* is a set (named *universe of discourse*) and *m* is membership function *m*:*U* → [0, 1] that associates each element *u* ∈ *U* to a real value ranging from 0 (the element *u* does not belong to set *U*) to 1 (the element *u* fully belongs to set *U*). Such function can have any form and shape, but it must return a value for any possible element in *U*; traditionally, fuzzy sets are implemented using triangular or trapezoidal membership functions. Fuzzy sets can be used to model and characterize linguistic variables, that is, objects or quantities of the real world that can be described using a fuzzy term. One example is the linguistic variable ‘temperature’, that could be described using the intrinsically fuzzy terms ‘cold’, ‘mild’ or ‘hot’.

Linguistic variables and fuzzy terms can be combined by means of traditional logic connectors (e.g. AND, OR, NOT) to formulate predicates about the world expressed in the form IF <antecedents> THEN <consequent>, where the overall degree of truth of the antecedent is calculated according to the membership degrees of the linguistic variables involved (the interested reader can find additional information in [7,38]). Such predicates take the name of *fuzzy rules* and can be used to model concepts of the world characterized by uncertainty. In fuzzy rules, the consequent is typically an output variable of the model to be predicted using one of the existing FISs. In this paper, we consider 0-order Takagi–Sugeno inference, in which the consequent takes the form <output_variable> IS <value>.

Fuzzy logic is a wonderful tool to help modelling phenomena that are characterized by intrinsic uncertainty and cannot be described in certain terms. The fuzzy rules, the linguistic values, the fuzzy sets and the shape of the membership functions are usually defined by the modeller, according to the available knowledge about the system to be described, unless a data-driven approach is exploited [39]. Fuzzy set theory and fuzzy logic found applications across several scientific and engineering disciplines, and FIS allowed fuzzy logic to be effectively adopted in several contexts, both in knowledge-based or data-driven applications, to support decision-making [40,41], meta-heuristics [42], modelling and control [43,44], clustering [45], classification tasks [46] and regression problems [47].

Thanks to the possibility of modelling different degrees of membership, this formalism provides a sound mathematical foundation to perform reasoning in conditions of uncertainty, handling vague concepts and connecting human language to numerical data.

As fuzzy logic allows to model vague concepts, it represents an excellent conceptual framework to model in-wake flying. In fact, air vortices have an intrinsic fuzzy nature as they do not have sharp boundaries, but they get gradually weaker in all the directions in which they develop. For this reason, it is also hard to clearly separate between areas being in-wake or not, even more when considering three different dimensions simultaneously.

### 2.4. A fuzzy model for in-wake flight characterization

To establish when a bird in the group is exploiting the upwash produced by another individual, five conditions must be satisfied. Both the conditions and then the parameters chosen for the model are knowledge-based; therefore, we reviewed the literature and extracted information about upwash and downwash development and in-wake flying.

Given a dyad of birds—one defined as the ‘leader’ and the other one as the ‘follower’—such conditions are:

(i) a follower can only fly in-wake when flying behind the leader, that is, the *n*|*s* axis component must have a negative value;
(ii) we assume that the follower can exploit the upwash, with respect to the *n*|*s* component, if its distance is neither too far to the leader nor too close [16,19,48,49];
(iii) considering the *e*|*w* axis, the follower must never fly directly behind the leader to avoid the downwash area. In fact, we suppose that in-wake flying is more efficient when one of the follower's wingtips is aligned with one of the leader's and less efficient otherwise. In particular, there is an optimal ‘wingtip overlap’, which is proportional to the birds' wingspan (average wingspan of northern bald ibis: *ca* 1.5 m) [15–17,19,26,27,49];
(iv) we also assume that the follower must be co-planar with the leader to exploit the upwash, i.e. the *u*|*d* values must be similar [17,25–27];
(v) finally, we suppose that the follower is able to exploit only a single upwash at the time.

All these conditions are intrinsically vague and gradual, and there is not a crisp boundary that can be used to determine when a condition is verified or not. A bird might satisfy each condition only partially.

**Figure 2.**
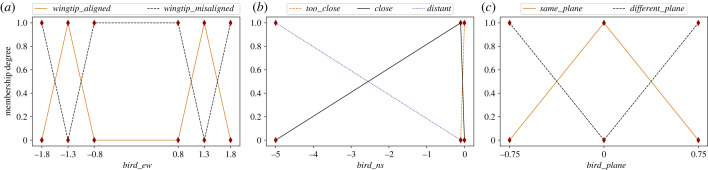
A representation of the three linguistic variables and the corresponding linguistic values and fuzzy sets, which define (*a*) lateral displacement (*bird_ew*), (*b*) anteroposterior distance (*bird_ns*) and (*c*) co-planarity (*bird_plane*) of the following bird with respect to the leader. Distance on the *x*-axis is measured in metres.

To still characterize these conditions in uncertain terms, we leverage a FIS to model in-wake flying. By using the relative positioning information among all the bird dyads as input, we implemented a 0-order Takagi–Sugeno FIS to calculate the extent to which the following bird is exploiting the upwash. To describe the aforementioned conditions, we defined three linguistic variables in the FIS, one for each dimension: (i) ‘*bird_ew*’, which models the follower's lateral displacement with respect to the leader (figure 2*a*); (ii) ‘*bird_ns*’, which corresponds to the distance of the follower on the anteroposterior axis (figure 2*b*) and (iii) ‘*bird_plane*’, which defines if the birds are co-planar (figure 2*c*).

The first linguistic term, ‘*bird_ew*’ (figure 2*a*), determines to what extent the follower's wingtip is aligned, or overlaps, with the wingtip of the leader. We modelled this variable with two fuzzy sets, which complement each other and are both symmetrical. The fuzzy sets are describing the alignment or overlapping (*wingtip_aligned*) or the misalignment (*wingtip_misaligned*). The membership function of ‘*wingtip_aligned*’ is maximum at ±1.3 m, when the two birds' wingtips are overlapping 20 cm. The membership value decreases to zero at ±1.8 m, when the birds’ wingtips are completely misaligned. The membership is also equal to zero when the follower is flying behind the leading bird, because of the downwash area [16,50]. Accordingly, the membership function of ‘*wingtip_misaligned*’ is equal to 1 when *e*|*w* ∈ [ − 0.8, 0.8] and for *e*|*w* > 1.8 and *e*|*w* < −1.8.

For the second linguistic variable, ‘*bird_ns*’ (figure 2*b*), we defined three fuzzy sets: ‘*too_close*’, ‘*close*’ and ‘*distant*’. Condition (i) states that the *n*|*s* baseline must be negative. Hence, the membership to all fuzzy sets drops to zero in the case of positive values. The membership function to the ‘*close*’ set increases between 0 and −0.1 m, where it reaches its maximum, then, it slowly decreases, reaching a value 0 when the follower is too distant from the leader (i.e. *n*|*s* < −5 m). The membership to the ‘*too_close*’ fuzzy set increases and decreases very fast between 0 and −0.1 m, modelling the circumstance in which the bird might crash into the leader if flying too close to the wing.

Finally, the last linguistic variable is ‘*bird_plane*’, which is defined by two complementary fuzzy sets ‘*same_plane*’ and ‘*different_plane*’ (figure 2*c*). In condition (iv), we assume that only a bird flying co-planar to the leader can exploit the upwash, so that the maximum value of the ‘*same_plane*’ membership is 1 at *u*|*d* = 0 m, and it declines for greater and smaller values. The membership drops to 0 at ± 0.75 m, which is when the follower flies half a wingspan higher or lower with respect to the leading bird.

For all fuzzy sets, the membership value remains constant outside the explicitly modelled universe of discourse (e.g. the membership to ‘*different_plane*’ is equal to 0 for any value *u*|*d* ∈ [ − ∞, − 0.75]). Once these linguistic variables had been defined, we drafted five fuzzy rules using the linguistic variables defined above as antecedents (table 1).

**Table 1.** Fuzzy rules used by the in-wake flying model.

Rule 1:	IF bird_ew IS wing_tip_aligned AND bird_ns IS close AND bird_plane IS same_plane THEN flying IS in_wake
Rule 2:	IF bird_ew IS wing_tip_misaligned THEN flying IS not_in_wake
Rule 3:	IF bird_ns IS too_close THEN flying IS not_in_wake
Rule 4:	IF bird_ns IS distant THEN flying IS not_in_wake
Rule 5:	IF bird_plane IS different_plane THEN flying IS not_in_wake

**Figure 3.**
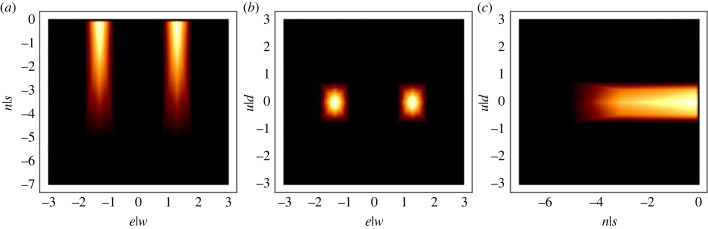
Heatmaps showing the mapping between relative positions and the defuzzified value of the output: (*a*) above, supposing that *u*|*d* = 0; (*b*) behind, with *n*|*s* = 1 and (*c*) from the side, assuming *e*|*w* = 0. The colour scale represents the strength of the ‘in_wake’ rule: the brighter, the stronger. Measurements are always in metres.

We defined two crisp output values for the consequents: in_wake = 1 and not_in_wake = 0. In figure 3, we used three heatmaps (one for each linguistic variable) to represent the behaviour of the FIS. The figures describe the three-dimensional space behind the leader and represent the level of upwash for the model: a brighter colour corresponds to a higher value of the in_wake output calculated by the FIS. The maximum region lies approximately 0.1 m behind the leading bird.

## 3. Results

### 3.1. Capturing the flock's dynamics

We tested our knowledge-based FIS on data collected from free-flying northern bald ibis. We implemented the FIS using the Python library Simpful, version 2.5.0 [51]. We ran the FIS on 10 min of relative position data for 29 birds, an average of 3004 datapoints for each individual (range 2997–3005). The model outputs a file for each bird considered as a follower. For each timestamp, it reports the identity of the leading bird and the relative coordinates of the follower with regard to the leader if the bird is in-wake. If the bird is not in-wake, it reports the relative coordinates of the nearest bird in the front, or zero if there is no bird in the front. Additionally, it yields a file reporting the strength of the fuzzy outputs.

**Figure 4.**
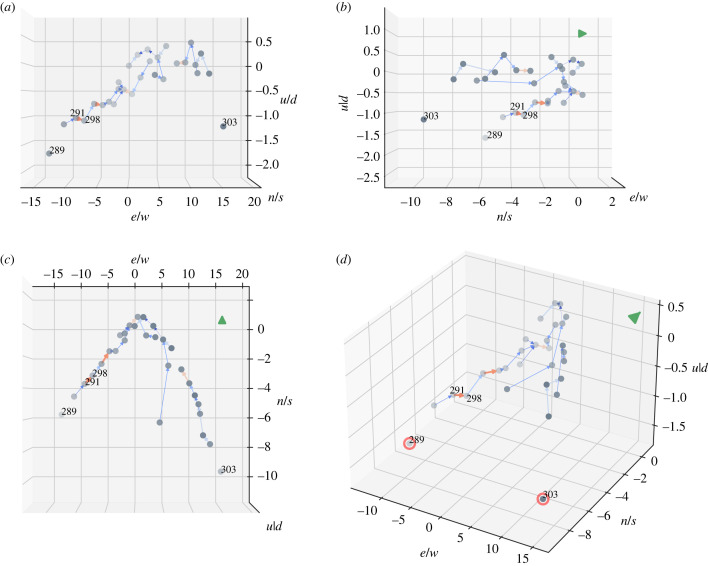
Example of in-wake relationships of the flock calculated by our FIS. View of the bird flock from different perspectives, i.e. back (*a*), the side (*b*), the top (*c*) and perspective view (*d*). The grey dots represent the positions of the birds, while the arrows denote the in-wake relationship between follower and leader bird. The colour of the arrow denotes the firing strength of the ‘in-wake’ rule, ranging from blue (low strength) to red (high strength). In (*d*), the red circles highlight the birds that are not flying in-wake. The green arrows show the direction towards which the entire flock is flying, except in (*a*), in which it is not present as the arrow goes inside the paper. For representation clarity, we report only the identity number of the birds mentioned in the text.

Thanks to these data, we can plot flock dynamics, and an example of a snapshot from different perspectives can be seen in figure 4. Birds are plotted with filled grey dots. When the FIS determines that a bird is in a good position to exploit the upwash, the circumstance is represented by an arrow and the colour of the arrow determines the strength of the fuzzy output, ranging from blue (low) to red (very high). For example, in figure 4, bird 291 is in a good position to exploit the upwash with respect to bird 298. The model can also calculate which birds are not flying in-wake: these are the grey dots without any outgoing arrow and, in figure 4*d*, they are highlighted by red circles (see, e.g. birds number 303 and 289 always in figure 4). In general, the different projections in figure 4 show that in this snapshot the flock was compact (figure 4*b*), all the birds were flying roughly co-planar (figure 4*a*) and disposed in a rough V-formation (figure 4*c*).

**Figure 5.**
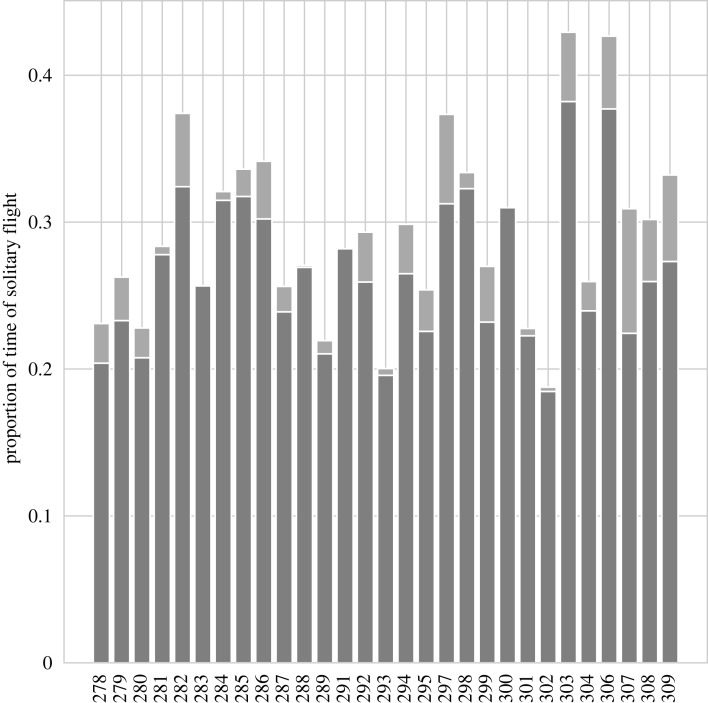
Proportion of the snapshots in which birds were flying alone. Darker grey defines when the birds were flying without being in the wake of any bird whereas the lighter grey is when they were flying with no other individual in front.

By analysing all snapshots corresponding to the 10 min flight, we can gain more insights into the flock's dynamics. First of all, we looked into bouts' duration both when birds were in-wake or not. Bouts were on average 1.10 ± 1.46 s long (median 0.6 s, range 0.2–23.4 s). In-wake bouts were on average 1.04 ± 1.39 s long (median 0.6, range 0.2–23.4 s), whereas not in-wake bouts were 1.23 ± 1.61 s long (median 0.8 s, range 0.2–18 s). Then, we calculated the proportion of the flight that each individual was flying alone, i.e. not in-wake. Alone flight can be due to two circumstances: either the bird was flying with no other individuals in the front, or it was not flying in the wake of any individuals (i.e. the output of the FIS is 0). According to our results, the birds flew 29.2 ± 7.5% of the time alone, ranging from 18.8% for bird 302 to almost 43% for bird 303 (figure 5).

**Figure 6.**
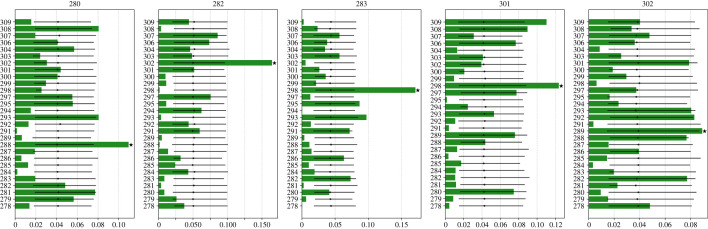
The green histograms show the proportions of in-wake flying behind a specific bird for five randomly chosen individuals. The identity of the bird flying in-wake is stated on top of each panel whereas the identification number of every other bird that plays as a leader on the *y*-axis. The *x*-axis reports the frequency of in-wake flying, the highest of which is denoted with a black star. The 95 per cent confidence intervals for expected values of following under the null-assumption of no individual preference (black bars) are based on non-parametric bootstrapped values.

From the snapshots where the birds were in-wake, we could calculate the distribution of the preferred leaders for each individual. Figure 6 presents this analysis for five birds, where each panel corresponds to a specific following individual (the histograms of the whole flock are reported in electronic supplementary material, figure S1). According to our results, each bird seems to fly preferably in the wake of one or few specific individuals. As an example, in figure 6, we notice that individual 280 flies mainly in the wake of 288, the most preferred and flagged by the black star, whereas 301 in the wake of 298 and 309. One of the birds, 282, flies more than 15% of the time behind bird 302 whereas in other two cases, the followers fly more than 10% of the time behind a specific individual (individual 301 behind 298 and 280 behind 288). Taking into consideration all the birds of the flock, the mean proportion of time that each bird spends behind every other is rather low, i.e. 0.037 ± 0.032. This result suggests also in this case that the birds had a preferred leader to follow (see electronic supplementary material, figure S1).

### 3.2. Identification of subgroups in the flock

**Figure 7.**
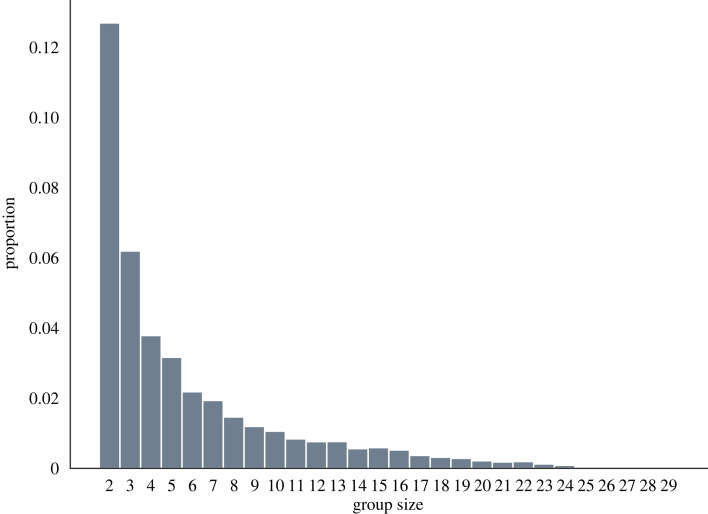
Distribution of subgroup sizes within the flock. Smaller groups are more common than bigger ones.

As shown in figure 4, we can determine whether a bird is exploiting the upwash provided by another individual for each snapshot of the flight. These relationships induce a directed acyclic graph, where the nodes represent the birds in the flock and two nodes (*j*, *k*) are connected by a directed edge (arc) if *j* is exploiting the upwash created by bird *k*. By performing a structural analysis on the resulting graph [52], we could calculate the weakly connected components to determine whether there were subgroups of ibises flying together, and the cardinality of such subgroups. For example, in figure 4*d*, we can affirm that individuals 291 and 298 are part of subgroups including 13 all-interconnected birds. As such, we could also quantify the size of all the subgroups to estimate birds' preference. Figure 7 shows the distribution of subgroup sizes. In accordance with a previous work from our group [27], birds preferred to fly in dyads (greater than 10% of flying time) or in triads whereas bigger groups are progressively less common. Despite the presence of these subgroups, in which the individuals can gain aerodynamic advantage, the entire flock remained cohesive (figure 4 and electronic supplementary material, video).

### 3.3. Comparison with frontal nearest neighbour

**Figure 8.**
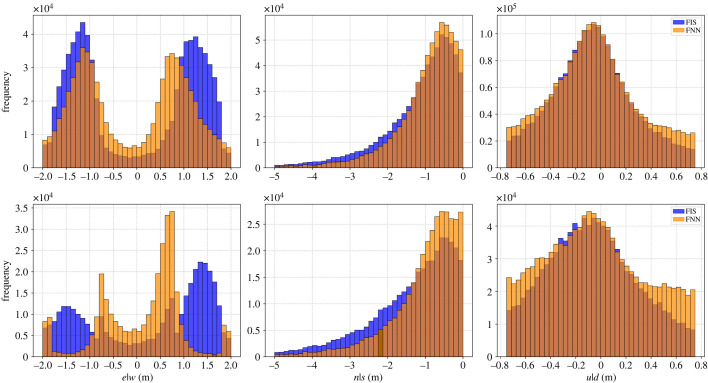
The histograms are showing the outputs of the FIS (in blue) and FNN (in orange) models in the three different dimensions considering the position of the follower with regard to the leader for all the timepoints. Histograms in the top row show all output points for both models, i.e. when they are in agreement and disagreement on the identity of the leading bird. To highlight differences, histograms in the bottom line show only the points in which the two models disagree. Data for all the birds pooled together.

We compared the results of the FIS and the FNN, specifically we investigated the leader identity determined by the two competing models (figure 8).

According to our results, the two models agreed on average in 47.6% of the cases (values ranging from 28.1 to 67% for different individuals). The top row of figure 8 reports a visual representation of the two models’ outputs (FNN in orange and FIS in blue) for all birds in the flock taking into account each axis separately. On the *e*|*w* baseline graph (top left), both models have a bimodal distribution with distinguishable peaks. The FNN model has its peaks at −1.3 and 0.8 m, whereas the FIS histogram peaks at −1.3 and 1.3 m. On the *n*|*s* baseline (top centre), both models have a right-skewed distribution, whereas on the *u*|*d* baseline (top right) they have a heavy-tailed normal distribution.

We further investigated the discrepancy between the two models by extracting only the snapshots in which the two models did not agree on the leaders' identity and by considering the position of the follower with regard to the leader. The bottom row of figure 8 shows graphically this dissimilarity. The distributions present a stronger disagreement, in particular in the case of the *e*|*w* component (bottom left panel). Both models follow a multimodal distribution; however, while in the FNN the modes are at approximately −0.8 and 0.8 m, in the FIS, the modes are at *ca* +1.5 and −1.5 m. This means that, when in disagreement, the FNN tends to yield solutions where the follower is flying progressively more behind the leader, a zone that should instead be avoided due to the expected downwash. On the *n*|*s* axis, we can observe a difference in the two models, in particular the FNN has a higher peak between 0 and 1 m, whereas the FIS output is characterized by a higher variance. Considering the *u*|*d* component, the two models’ output do not extremely differ, although the FIS tends to favour co-planarity.

As such, these visual results highlight that the difference between the two models is mainly a consequence of the output distributions on the *e*|*w* component. On a large dataset, this leads to complete different results. These analyses confirm that the FNN model, by definition, privileges the nearest frontal leader, so that the output tends to be biased towards the closest individual regardless the regions of downwash and upwash.

## 4. Conclusion and future developments

We successfully modelled in-wake flying of northern bald ibises by using fuzzy logic. Specifically, we defined a FIS with three linguistic variables, one for each spatial dimension, and devised five fuzzy rules. We showed that, thanks to this FIS, we can reconstruct flock dynamics, identify subgroups inside the flock, recollect some insight on social relationships in the flock and, most importantly, determine the in-wake state of each bird. With the model, we analysed a 10 min flight of a flock of 29 birds.

During the flight, the flock was quite dynamic as birds changed their positions quite often, as suggested by the short duration of the bouts. Moreover, the birds did not form a big unique formation, but arranged in small subgroups of individuals, mainly dyads and triads. This evidence is in line with former results of our group, where Voelkl *et al*. [27] reported that bouts of continuous in-wake flying were rather short [27]. Furthermore, they stated that the birds preferred to fly in small groups, which is common also when they are migrating in the wild ([53], personal communication). Extracting subgroups within a flock might be important, for example, to study phenotypic variation, individual heterogeneity and group dynamics within the flock. In fact, several studies in different taxa reported that individuals inside a group vary in their physical and physiological characteristics, and therefore they have to find a *consensus* to remain together [54,55]. For example, flocks of homing pigeons (*Columba livia*) are formed by individuals with different mass, which is correlated with their travel speed. When flocking, larger individuals needed to fly slower, to allow smaller individual to keep up [56]. In moving groups of wild olive baboons (*Papio anubis*), individuals compromised on gait and pauses to favour group cohesion [57]. This heterogeneity among group members might lead to the formation of subgroups. Subgroups might also be important when exploring social relationships. In our data, although birds changed their position often, they seemed to prefer flying in the wake of specific individuals. The reasons of this behaviour might be a cooperation mechanism based on kin selection or reciprocation. In our flock of northern bald ibises, some birds were related and raised in the same nest, some related individuals were divided in different nests and some were not related but raised together. However, when considering relatedness, raising time and preference of flying together, we saw no particular pattern. The favourite bird of each individual was rarely a brother or a nest-mate, which instead they seemed to avoid. For example, bird 308 had a brother, bird 309, and was in the nest with it and other two birds, 303 and 306. The ‘favourite mates’ of 308 are instead individuals 279 and 292 (figure 6), which suggests that the mechanisms involved might be reciprocity, as already proposed by Voelkl *et al*. [27].

The most important output of our model is defining the state of the bird, hence, to classify if it is in-wake or not. Some past attempts used the FNN to do this classification, but we have shown that the outputs of the FIS and the FNN strongly disagree. The FNN is based on Euclidean distance and does not take into consideration the position of the air vortices. Thus, the FNN might often return solutions that are aerodynamically disadvantageous based only on vicinity. The FIS, instead, tries to model closely where these vortices lie. Studies on *cluster formations* do not need to consider these air vortices as birds change their flight direction and their position in the flock very rapidly. However, it is a fundamental aspect when investigating line formation and energy savings. In fact, we have to cautiously distinguish when the bird is flying in-wake to then correlate this state with measurements of energy expenditure. To the best of our knowledge, we are aware of a single empirical study with pelicans that tried to relate heart rate with in-wake flying [18]. The lack of studies in this area does not reflect the scarce interest of scientists in the topic but highlights the challenge in collecting empirical data and in modelling in-wake flying. With our study, we want to start reducing this lack of knowledge in the field, by creating a model that aims to decrease the error in categorizing in-wake flying. In literature, there are already some attempts to use fuzzy logic to model bird flocks. For example, in [7], the authors modelled *animats* and their drives using fuzzy logic and observed the development of flocking behaviour. As a further step, in [28], the authors tried to unveil the evolution of collective behaviour introducing predation pressure. However, these models differ greatly from our conception for three main reasons: (i) due mainly to technical challenges to collect data, all these investigations were never calibrated against empirical data, (ii) their perspective was always on single individuals, trying to model their behaviour to study the emergence of flight formations, and (iii) the fuzzy-based simulations focused almost exclusively on the investigation of *cluster formations* and seldom considered *line formations*.

We are aware that the output presents high-frequency changes, for example, a following bird might fly in-wake of an individual to then switch very shortly to another (200/400 ms) and then back to the first one or switch again to another. These frequent changes have been described in previous work [27] and result from the fact that birds flew not only in a line formation but also in a cluster. Therefore, individuals were rather close to each other and their relative position to any other bird in the flock could change fast and frequently. However, the frequency of such changes might have been overestimated by the parameters that we used to specify the membership functions. To reduce this problem in future studies, one could limit extraction to bouts of in-wake flying that have a minimum time duration.

Finally, our model does not account for flapping synchronization between the leading and the following bird. Previous studies suggested that, in order to save energy, a bird flying in-wake should synchronize the flapping cycle with the bird providing the upwash, considering also the phase shift due to the distance between the two birds [58]. However, about this phenomenon there is scarce and contrasting evidence. Portugal *et al*. [16] reported that northern bald ibises synchronize their flapping; however, no effect was found when investigating shorebirds mixed-species flocks [26]. In addition, to the best of our knowledge, there is no study proving that flapping synchronization plays a role in saving energy. Given this contradictory evidence, we did not include this aspect in the model, but we assume that, if the following bird is in the right position to exploit the upwash, it is also able to feel the air vortex and to synchronize with the leading bird.

Future studies should use this model to start studying more in depth in-wake flying and energy savings. The first step would be to collect and correlate any type of energy expenditure measurement (dynamic body acceleration, heart rate, double labelled water, etc.) with birds' state inside the formation (in-wake or not) to try to quantify how much energy birds save. Then, it would be interesting to study the different types of formation, as for example line and V-formation, and investigate whether they are used interchangeably by the birds or if a type of formation is preferred when flying over a specific environment or when there are specific weather conditions. Flock dynamics and individual heterogeneity could also be of particular interest, to unveil if all the birds participate to line formation and if all of them occupy all the positions inside the formation. Specifically, flying in the front of the formation is not advantageous, so individuals should not be inclined to be in this position much longer than other individuals. Finally, future studies could look into social relationships among birds and how those influence line formation, such as the pattern of exchange of positions in the formation. Birds flying closely together or in the same formation could be socially associated [59], which would allow to build social networks, and possibly comparing those with measures of relatedness and social networks built on associations happening when observed at the ground.

To conclude, we have implemented a novel model based on fuzzy logic for line formation and performed a first test on empirical data. Future application on larger datasets will help to unravel even more aspects of line formation, so far poorly understood due to the lack of empirical data.

## Data Availability

All data is available at the link: https://phaidra.vetmeduni.ac.at/o:1287 [60]. The data are provided in the electronic supplementary material [61].

## Endnotes

1 More information available at: http://waldrapp.eu.

2 Data available at: https://igs.bkg.bund.de/root_ftp/EUREF/obs/2019/220/PFA300AUT_R_20192200000_01D_30S_MO.crx.gz.
